# Chemical Constituents and Antifungal Properties of *Piper ceanothifolium* Kunth Against Phytopathogens Associated with Cocoa Crops

**DOI:** 10.3390/plants14060934

**Published:** 2025-03-16

**Authors:** Yudy S. Mahecha-Jimenez, Oscar J. Patiño-Ladino, Juliet A. Prieto-Rodríguez

**Affiliations:** 1 Departamento de Química, Facultad de Ciencias, Universidad Nacional de Colombia (UNAL), Sede Bogotá, Bogotá 111711, Colombia; ymahechaj@unal.edu.co (Y.S.M.-J.); ojpatinol@unal.edu.co (O.J.P.-L.); 2 Departamento de Química, Facultad de Ciencias, Pontificia Universidad Javeriana (PUJ), Sede Bogotá, Bogotá 110231, Colombia

**Keywords:** *Piper ceanothifolium*, *Theobroma cacao*, *Moniliophtora roreri*, *Lasiodiplodia theobromae*, *Fusarium solani*, prenylated hydroquinones, chromene

## Abstract

In this study, the antifungal potential of chemical constituents of *Piper ceanothifolium* Kunth was determined against three phytopathogenic fungi associated with the cocoa crop. The methodology included the phytochemical study of the inflorescences of *P. ceanothifolium*, the synthesis of a chroman-4-one type derivative and the evaluation of the antifungal activity against *Moniliophthora roreri*, *Fusarium solani*, and *Lasiodiplodia theobromae*. The phytochemical study led to the isolation and identification of two new hydroquinones (**1** and **5**), together with three known compounds (hydroquinones **2** and **3,** and chromene **4**). The synthesis of a new chromone **6** obtained from **2** through an oxa-Michael type intramolecular cyclization is also reported. All compounds showed strong antifungal activity, with **6** (IC_50_ of 16.9 µM) standing out for its action against *F. solani*, while prenylated hydroquinones **1** (30.4 µM) and **2** (60.0 µM) were the most active against *M. roreri* and *L. theobromae*, respectively. The results of this research represent the first report of the chemical composition and antifungal properties for *P. ceanotifolium*, suggesting its potential use as a control method against *M. roreri*, *F. solani*, and *L. theobromae*.

## 1. Introduction

*Theobroma cacao* L., one of the most representative species of the Malvaceae family, is a source of various useful raw materials for the food, pharmaceutical, and cosmetic industries [1,2]. At the therapeutic level, it is characterized for its stimulating effect on the central nervous system and for its antioxidant, antibacterial, antipyretic, antianemic, antihypertensive, and anti-inflammatory properties [3,4]. Both the production and export of cacoa are tools for strengthening and increasing competitiveness in the agricultural sector for underdeveloped countries, so it is considered a product of great socioeconomic value [5,6]. On the other hand, according to the International Cocoa Organization, the demand for cocoa beans has increased by 2.5% per year, which allows for the forecast of a deficit of two million tons of cocoa by 2030. In addition, production yields are moderate to low in some regions due to several factors, including infestation by various pests [7,8,9].

Fungi and oomycetes are important phytopathogens associated with cocoa crops, for which it has been reported that they can reduce production yields by 30 to 80% [10,11]. These phytopathogens cause various diseases in cocoa plantations, altering their physiological state, and in extreme cases causing the death of the plant [10]. Among the most representative are, *Moniliophthora roreri*, which affects the fruits and causes the disease known as moniliasis [11,12], *M. perniciosa*, that affects the growing tissues of the plant causing the disease known as “witches broom” [13,14], and, *Ceratocystis fimbriata*, which causes the disease known as “machete disease” that affects the vascular system and can cause the death of the plant [15,16]. In addition, secondary pests have been reported, such as the *Phytophthora* species, which attack the fruit and vascular system and cause the disease known as “black ear” [17], and *Fusarium solani* and *Lasiodiplodia theobromae*, which attack the vascular system and cause the disease known as “vascular streak dieback” (VSD) [18,19]. There are different strategies for the control of these phytopathogens that involve integrated management of the crop and the use of commercial pesticides [20,21]. Although these chemicals are effective, many of them have been associated with environmental damage and health problems for farmers and consumers. In this way, it is necessary to develop new effective and safe products to control this type of pest.

Thus, plants can be considered as a potential alternative due to the great variety of specialized metabolites that they produce in response to different biotic and abiotic factors, and for which their properties for pest control have been determined [22,23,24]. The genus *Piper*, which belongs to the family Piperaceae, is recognized for the antifungal potential of its extracts, essential oils, and chemical constituents, which can be used for the control of phytopathogens that affect agricultural crops of commercial importance. Among the specialized metabolites described for the genus *Piper* that stand out for their antifungal properties are alkylbenzenes, amides, benzoic acid derivatives, phenylpropanoids, flavonoids, and terpenes [25,26,27,28,29,30,31,32,33]. There are very few studies on species of the genus *Piper* for the control of phytopathogens associated with cocoa cultivation, but generally there are reports on the preliminary antifungal potential of some extracts and essential oils against *Phytophtora palmivora*, *P. capsici*, *M. roreri*, *L. theobromae*, and *F. solani* [29,30,31,32,33]. Research on chemical constituents present in bioactive species is not very common, highlighting the study carried out on *P. pesaresanum* where the antifungal properties of the extract, fractions, and chemical constituents against *M. roreri*, *Phytophtora* sp., and *F. solani* were determined. In addition, some preliminary structure–activity relationships were determined from the most active constituents [33].

*Piper ceanothifolium* Kunth (synonyms *Enckea ceanothifolia* (Kunth) Kunth, *P. amalago f. ceanothifolium* (Humb., Bonpl. & Kunth) Steyerm., *P. amalago* var. *ceanothifolium* (Kunth) Yunck., and *P. medium* var. *ceanothifolium* (Kunth) Trel. & Yunck), is a native species distributed in tropical countries such as Colombia, Panama, Venezuela, and Brazil [34,35,36]. There are no reports in the literature on the chemical composition and biological properties for this species. The present research describes the antifungal potential of chemical constituents from *P. ceanothifolium* against three phytopathogenic fungi associated with the cocoa crop (*M. roreri*, *M. perniciosa*, and *L. theobromae*).

## 2. Results and Discussion

### 2.1. Phytochemical Study and Inhibition of Mycelial Growth Assays

The fractionation of ethanolic extract (EE) from *P. ceanothifolium* inflorescences using vacuum liquid chromatography (VLC) led to four fractions of different polarity being obtained (dichloromethane (DCM), ethyl acetate (EtOAc), isopropanol (IPA), and ethanol/water 8:2 (EtOH:H_2_O 8:2). EE and each of the fractions obtained were subjected to inhibition of mycelial growth (IMG) assays against *F. solani*, *L. theobromae*, and *M. roreri*. The results obtained show that the antifungal activity on the three fungi was concentrated in the DCM fraction (Table 1). The DCM fraction in comparison to the EE presented lower IC_50_ against *L. theobromae* and *M. roreri*, while on *F. solani* no significant differences were found. In this way, the DCM fraction was selected to undergo purification by chromatographic techniques to isolate and identify the bioactive constituents.

**Figure 1:**
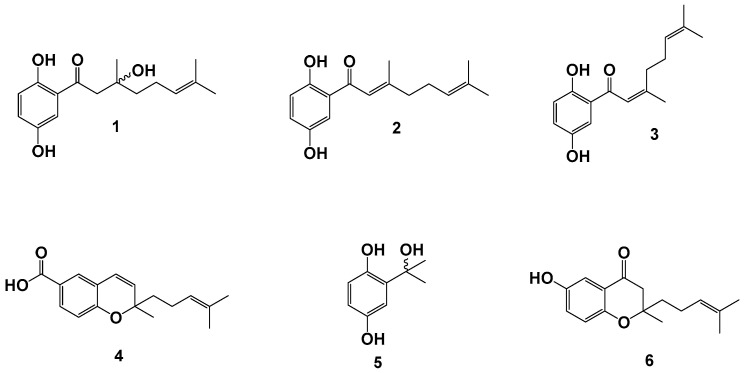
Chemical constituents from *P. ceanothifolium* (**1**–**5**) and a synthesized derivative (**6**).

The phytochemical study carried out on the DCM fraction from *P. ceanothifolium* led to the isolation and identification of two new hydroquinones (ceanothifolione (**1**) and ceanothifoliol (**5**)), along with three known compounds (debromocymopolone (**2**)**,** 1,4-dihydroxy-2-(3′,7′-dimethyl-1′-oxo-2′-*Z*-6′-octadienil)benzene (**3**) and lhotzcromene (**4**)). These chemical constituents are reported for the first time in the species; however, compounds **2** to **4** have been previously reported in other species of the *Piper* genus [37,38,39,40,41]. The structural characteristics of **1**–**5** are in accordance with the chemotaxonomy of the genus since some of its species have been reported to have prenylated hydroquinones with oxogeranyl chains and prenylated derivatives of cyclic benzoic acid (chromenes) [25,29,42]. On the other hand, chroman-4-one **6** was synthesized for the first time and in good yield from **2** through an intramolecular cyclization of oxa-Michael type with some modifications [43]. Figure 1 shows the chemical structures for the isolated and synthesized compounds.

Compound **1** was obtained as a white solid with a m.p. of 82–83 °C, which produces a dark brown color when sprayed with the FeCl_3_ reagent on TLC, indicating the possible presence of phenolic hydroxyls [44]. According to the NMR analysis (Figure S1 and Figure S2 in Supplementary Materials), it was determined that **1** presents a 1,2,4 trisubstituted aromatic ring by the signals in ^1^H with δ_H_ 7.39 (d, *J* = 3.2 Hz, 1H), 7.09 (dd, *J* = 8.9; 3.2 Hz, 1H), and 6.84 (d, *J* = 8.9 Hz, 1H), together with the signals in APT at δ_C_ 154.2 (C), 150.1 (C), 125.4 (CH), 120.2 (C), 119.6 (CH), and 110.6 (CH). Additionally, it was determined that the substituents on the aromatic ring correspond to two hydroxyl groups (signals in APT with δ_C_ 154.2 (C) and 150.1 (C)) and a prenylated chain of the geranyl type (3′-hydroxy-3′,7′-dimethyl-1′-oxo-6′-octenyl) which is characterized by having a carbonyl group in the 1′ position and in the 3′ position a hydroxyl group. This prenylated substituent has been previously reported in the literature and is characterized spectroscopically by the signals at δ_H_ 5.06 (t, *J* = 7.2, 1H), 2.78 (d, *J* = 16.7, 1H), 2.66 (d, *J* = 16.7, 1H), 2.12–2.08 (q, 2H), 1.83–1.76 (m, 1H), 1.67 (s, 3H), and 1.57 (s, 3H), and the signals with δ_C_ 194.0 (C), 132.3 (C), 123.3 (CH), 80.8 (C), 47.4 (CH_2_), 39.0 (CH_2_), 25.6 (CH_3_), 22.2 (CH_2_), and 17.6 (CH_3_) [37,41,45]. The use of two-dimensional experiments (COSY, HMQC, and HMBC, see Figures S3–S5 in Supplementary Materials) allowed for the confirmation of the substructures and the location of the substituents on the aromatic ring. Using high-resolution mass spectrometry analysis (HR-MS) in positive mode, the molecular formula was established as C_16_H_22_O_4_ (*m*/*z* 278.1462 [M + H]^+^, calculated for C_16_H_22_O_4_, 278.1518) and its formula is consistent with the result of spectroscopic analysis. Thus, compound **1** is reported for the first time and we have named it ceanothifolione.

Compound **2** has an NMR profile like **1**, where the characteristic signals for prenylated hydroquinone are observed (Figure S6 and Figure S7 in Supplementary Materials). The difference in their spectra occurs in the prenylated chain, being for **2** of the oxo-geranyl type by the signals at δ_H_ 6.68 (s, 1H), 5.13 (t, *J* = 7.0 Hz, 1H), 2.30–2.24 (m, 4H), 2.20 (s, 3H), 1.73 (s, 3H), and 1.64 (s, 3H), and the signals at δ_C_ 196.8 (C), 161.9 (C), 133.6 (C), 124.9 (CH), 119.8 (CH),115.7 (CH), 42.3 (CH_2_), 26.9 (CH_2_), 26.4 (CH_3_), 20.8 (CH_3_), and 17.9 (CH_3_). Based on this analysis and by comparison with the spectroscopic data described in the literature, **2** was identified as debromocymopolone. This compound was isolated for the first time in the algae *Cymopolia barbata* and from there it derives its name [41]. In the Piperaceae family it has been reported in the leaves and roots of *P. crassinervium* [40]. Debromocymopolone **2** has been reported to have antiparasitic activity against *Trypanosoma cruzi* (IC_50_ of 6.1 µg/mL) and moderate antioxidant activity by inhibiting lipoperoxidation (IC_50_ of 26.43 µM) [37]. Antifungal activity against *Cladosporium cladosporioides* and *C. sphaerospermum* has been reported for **2**, with a minimum inhibitory quantity (MIQ) of 1.0 µg to inhibit both phytopathogens [38].

Compound **3** exhibits an NMR profile characteristic of prenylated hydroquinones, like compounds **1** and **2** (Figure S8 and Figure S9, Supplementary Materials). A comparison between **3** and **2** revealed comparable shifts in both the ^1^H and APT spectra, except for the signals corresponding to positions 4′ and 10′, suggesting that 3 could be a geometric isomer of **2**. NOESY analysis confirmed the relative positioning of substituents on the double bond in both compounds (Figure S10, Supplementary Materials). Based on these findings and a comparison with previously reported spectroscopic data, compound **3** was identified as 1,4-dihydroxy-2-(3′,7′-dimethyl-1′-oxo-2′Z,6′-octadienyl) benzene, a compound previously described in the leaves and roots of *P. crassinervium* [44]. The antifungal action of **3** against *C. cladosporioides* and *C. sphaerospermum* (MIQ of 5.0 µg and 10.0 µg, respectively) has been described in the literature [38]. Additionally, antioxidant properties have been reported due to its ability to inhibit lipoperoxidation (IC_50_ of 63.11 µM) [40].

Compound **4** was obtained as an amorphous solid with a m.p. of 48–49 °C, which produces a dark blue color when sprayed with the vanillin/H_2_SO_4_ reagent on TLC. That is characteristic for chromenes present in species of the genus *Piper* [29,44]. Analysis of the NMR signals for **4** (Figure S11 and Figure S12 in Supplementary Materials) confirms the presence of a prenylated chromene, which was identified as lhotzchromene and which was previously reported in the leaves of *P. lhotzkyanum* and roots of *P. crassinervium* [45]. There are no previous reports in the literature on the biological properties of lhotzchromene.

Compound **5** was isolated as a yellow crystalline solid with a m.p. at 53–55 °C, and by analysis of its NMR spectra (Figure S13 and Figure S14 in Supplementary Materials) the presence of a 1,2,4-trisubstituted aromatic ring was determined (signals with δ_H_ 7.31 (d, *J* = 3.0 Hz, 1H), 7.11 (dd, *J* = 8.9, 3.0 Hz, 1H), and 6.82 (d, *J* = 8.8 Hz, 1H), and with δ_C_ 155.6 (C), 149.3 (C), 124.8 (C), 119.4 (CH), 118.5 (CH), and 115.5 (CH)). The substituents on the aromatic ring were determined as two hydroxyls located in positions 1 and 4 (signals in δc 155.6 (C) and 149.3 (C)) and a group of type 1′-hydroxy-1′-methylethyl located in position 2, (δ_H_ 2.86 (s, 3H), 2.63 (s, 3H) and δ_C_ 88.6 (C), 29.6 (CH_3_), 26.0 (CH_3_)). Thus, **5** is reported for the first time, and we have called it ceanothifoliol.

**Figure 2:**
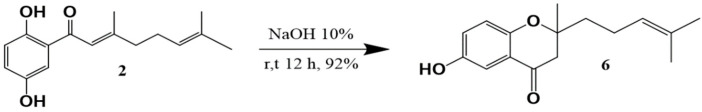
Synthesis of ceanothichromone (**6**).

Starting from the major compound debromocymopolone **2**, the chromone called ceanothichromone **6** was synthesized for the first time and with good yield, using typical conditions of an intramolecular cyclization of the oxa-Michael type (Figure 2) [43]. For the reaction carried out, the presence of a phenolic hydroxyl and a chain with *α*,*β*-unsaturated carbonyl was used for the formation of chromone-type compounds of which there are previous reports in the literature of their antifungal potential [37,38,39,40]. Compound **6** was characterized in NMR by the characteristic signals of the chromone base nucleus with δ_H_ 7.38 (s, 1H), 7.09 (dd, *J* = 9.1, 3.1 Hz, 1H), 6.87 (d, *J* = 9.1 Hz, 1H), and 2.80 (d, *J* = 16.7 Hz, 1H), together with the signals at δ_C_ 194.8 (CO), 157.1 (C), 154.5 (C), 125.0 (CH), 120.3 (C), 119.6 (CH), 110.7 (CH), 80.8 (C), and 47.5 (CH_2_). Additionally, some two-dimensional experiments (COSY, HMQC, and HMBC) were used for the proposal of each signal and the confirmation of the proposed structure (Figures S15–S19 in Supplementary Materials).

### 2.2. Antifungal Potential of Chemical Constituents of P. ceanothifolium

The antifungal potential of the chemical constituents **1**–**6** was determined by evaluating its inhibitory capacity on mycelial growth, fungicide–fungistatic effect, and inhibition of conidia germination against *M. roreri*, *L. theobromae*, and *F. solani*

#### 2.2.1. Inhibitory Capacity on Mycelial Growth

The results of antifungal activity expressed as half-maximal inhibitory concentration (IC_50_) are summarized in Table 2. The results show that compound **5** was the only one that did not cause any inhibition on the three fungi evaluated. The IC_50_ values for active compounds against the three fungi range from 16.9 μM to 199.2 μM. These results constitute the first report of antifungal activity against *M. roreri*, *L. theobromae*, and *F. solani* for all evaluated compounds (**1** to **6**).

The results of antifungal activity against *F. solani* show that chromenes **4** and **6** are the most active. Comparing the IC_50_ values of compounds **1**, **2**, and **3**, it is observed that the presence of a double bond at position 2′ of the aliphatic chain of prenylated hydroquinones, forming an α, β-unsaturated carbonyl system, increases the antifungal activity. In addition, the geometry of the double bond has a significant effect as the *E* double bond enhances the antifungal activity, making compound **2** twice as active as compound **3**. It is also found that the presence of the prenylated chain in hydroquinones is necessary to exert the antifungal activity, which is confirmed by the inactivity of compound **5** against *F. solani*. Furthermore, it can be concluded that the formation of a chromone significantly improves the antifungal activity against this phytopathogen when comparing the activity of compound **6** with its precursor **2**, the latter being twice as inactive. These results are consistent with previous studies on compounds from *Piper* species, which have shown that prenylated chromenes and chromones tend to exhibit greater antifungal activity [29,32,38].

The results of antifungal activity against *L. theobromae* show that the prenylated hydroquinones **1** and **2** were the most active substances in inhibiting the growth of *L. theobromae*, with **1** being approximately twice as active as **2**, which allows us to conclude that the presence of the hydroxyl group in the 3′ position of the prenylated chain has a positive effect on the antifungal activity. It is also observed that compound **2** is approximately twice as active as its isomer **3**, demonstrating that the *E*-isomerism of the double bond at the 2′ position allows better antifungal effects, which has also been observed for *F. solani*. Furthermore, it is evident that cyclic compounds of the chromene **4** and chromone **6** type are not as promising compared to the results obtained against *F. solani*. In the case of the open-chain benzoic acid, compound **1** is the only one that does not present significant differences with respect to mancozeb, so it can be considered the one with the greatest potential against *L. theobromae*.

The results of antifungal activity against *M. roreri* indicate that the most active compound was prenylated hydroquinone **2**, followed by its *Z*-isomer (compound **3**). Prenylated hydroquinones with a double bond at the 2′ position of the oxo-geranyl chain (compounds **2** and **3**) proved to be the most active, highlighting the importance of this double bond for antifungal activity against *M. roreri*. Like other pathogens, the *E* isomer was more active than the *Z* isomer. Chromene 4 was evaluated for the first time against this pathogen; however, a previous study by Chitiva et al. [32] identified two chromenes with activity comparable to mancozeb against *M. roreri*. One of these compounds was the chromene known as 2,2-dimethyl-8-(3′,3′-dimethylallyl)-2H-1-chromene-6-carboxylic acid (IC_50_ = 2.9 µM), a positional isomer of compound **4** that differs in the position of the isopentenyl group. The chromene evaluated by Chitiva et al. [32] was approximately 70 times more active than compound **4** (IC_50_ = 199.2 µM), suggesting that the position of the prenyl chain has a significant impact on the antifungal activity of these compounds. None of the compounds tested achieved inhibition levels like the positive controls, and *M. roreri* was the least susceptible fungus to treatment with these compounds.

#### 2.2.2. Fungicide–Fungistatic Effect

The fungicidal or fungistatic activity of the compounds with inhibitory potential against the three microorganisms studied was evaluated using a maximum concentration of 100 μg/mL. The prenylated hydroquinones (**1** to **3**), the chromene (**4**), and the chromone (**6**) showed a fungistatic effect, while the controls (mancozeb and benomyl) showed a fungicidal effect against the three phytopathogens evaluated (see Figure S21 in the Supplementary Materials). The specific mechanism responsible for the fungicidal activity has not yet been determined, although some authors have linked it to the inhibition of ergosterol, chitin, and/or glucan biosynthesis in the fungal cell wall as these components are essential for cell structure and are considered key inhibition sites in fungi [46,47]. This study represents the first analysis of the fungicidal–fungistatic effect of the five bioactive compounds (**1** to **4**, and **6**) on *M. roreri*, *F. solani*, and *L. theobromae* and contributes to the knowledge of the post-treatment effects of the evaluated compounds.

#### 2.2.3. Inhibition of Conidia Germination

Conidia are the main vector for fungal dissemination to their host [47,48,49]; therefore, it is essential to evaluate the ability of the compounds to inhibit their germination. To this end, a conidial germination inhibition assay was performed using the compounds with the highest antifungal potential (**1** to **4**, and **6**). The results, expressed as the percentage inhibition of conidia germination (% ICG), ranged from 25.5% to 85.2% (Table 3), indicating that the inhibitory capacity of the compounds also affects the reproductive structures of the fungi. Specifically, compound **6** was the most effective in inhibiting conidial germination in *F. solani* and *L. theobromae*, while compound **1** showed the best results in *M. roreri*. Although none of the compounds exceeded the inhibition percentage achieved by the positive controls at the concentration evaluated (IC_50_), most compounds were able to reduce conidial germination by more than 50% in all three microorganisms. This study represents the first report on the inhibition of conidial germination in *M. roreri*, *F. solani*, and *L. theobromae* of the evaluated compounds.

## 3. Materials and Methods

### 3.1. General Experimental Procedures

Vacuum liquid chromatography (VLC) was performed on SiliaPlate^TM^ silica gel F_254_ of size 5–20 μm (SiliCycle^®^ Inc., Quebec, QC, Canada). Flash chromatography (FC) was performed on SiliaFlash^®^ silica gel P_60_ of size 40–63 μm (SiliCycle^®^ Inc., Quebec, QC, Canada). Thin-layer chromatography (TLC) was performed on SiliaPlate^TM^ alumina plates pre-coated with silica gel 60 F_254_ (SiliCycle^®^ Inc., Quebec, Canada). The solvents used in the chromatographic techniques were of technical grade and were distilled before use. Melting points (m.p.s) were recorded on a Thermo Scientific 00590Q Fisher-Johns apparatus (Thermo Scientific^®^, Waltham, MA, USA). The ^1^H-NMR and APT, and 2D (COSY, HMQC, HMBC and NOESY) spectra were performed on a Bruker Advance AC-400 spectrometer (Bruker^®^, Leipzig, Germany) for the ^1^H-NMR and APT experiments, operating at 400 MHz for ^1^H-NMR and 100 MHz for APT. For high-resolution mass spectrometry analysis (HRMS), an LC-MS-TOF system (Shimadzu^®^, Duisburg, Germany) was used. The ionization method was operated with ESI in positive ion mode.

### 3.2. Strains and Fungal Growth Conditions

The fungi used for the bioassays were isolated from the organs of cacao plants with symptoms of vascular diseases. The molecular characterization of the fungi was carried out using the Sanger technique using the general primers ITS1 and ITS4. The sequences were compared with those reported in GenBank, finding that they have a 99% identity with *Moniliophtora roreri*, *Lasiodiploidea theobromae*, and *Fusarium solani*. The fungi were cryopreserved in 25% glycerol in cryovials at −80 °C, and for each bioassay they were activated following the methodology reported in the literature [47,48]. The strains were used after 5 days of growth for *F. solani*, 15 days for *M. roreri*, and 8 days for *L. theobromae* (Figure S21).

### 3.3. Plant Material

The inflorescences of *P. ceanothifolium* Kunth were collected in the rural part of the El Colegio municipality located in the department of Cundinamarca, Colombia (4.37392, −74.40591). The determination of the species was made by the biologist Ricardo Callejas, and a specimen has been deposited in the Herbario de la Universidad de Antioquia (HUA) with the collection number 217583.

### 3.4. Extraction and Isolation

The dried and ground inflorescences of *P. ceanothifolium* (450 g) were subjected to extraction with 96% ethanol using the maceration method at room temperature. In the extraction process, enough solvent was placed to cover the sample, and four extractions were carried out with changes of solvent every third day. The resulting solution was concentrated by distillation under reduced pressure to obtain 75 g of ethanolic extract. A part of the extract (74 g) was fractionated by VLC using solvents of different polarity: dichloromethane (DCM), ethyl acetate (EtOAc), isopropanol (IPA), and an ethanol–water mixture (EtOH:H_2_O 80:20). After evaporation of the solvents under reduced pressure, the fractions DCM (40 g), EtOAc (11 g), IPA (8 g), and EtOH-H_2_O (1 g) were obtained. These fractions were evaluated in the mycelial inhibition assay against *F. solani*, *M. roreri*, and *L. theobromae*, which led to the determination that the DCM fraction is active against both phytopathogens. The DCM fraction (40 g) was subjected to FC using a mixture of hexane/AcOEt with increasing polarity (80:20 to 50:50) as the mobile phase, which led to us obtaining six fractions (**1**–**6**). Fractions 1 and 2 were combined (29.4 g), and through successive FC with different mixtures of hexane/DCM/AcOEt (80:10:10; 70:15:15; 50:25:25), allowed the isolation of a white crystalline solid (**1**, 149 mg, m.p. 82–83 °C), an orange crystalline solid (**2**, 1.6 g, m.p. 74–75 °C), and a yellow oil (**3**, 215 mg). The pooled fractions of 3 and 4 (9.3 g) were subjected to FC with hexane/DCM/AcOEt (60:20:20), allowing the isolation of a white solid (**4**, 281 mg, m.p. 48–49 °C). Fractions 5 and 6 were combined (5.5 g) and subjected to FC using hexane/AcOEt (50:50) as the mobile phase, which led to the isolation of a yellow crystalline solid (**5**, 22 mg, m.p. 52–53 °C). The general purification diagram carried out on the DCM fraction from *P. ceanothifolium* is illustrated in Scheme S1 of the Supplementary Materials.

Ceanothifolione (**1**): A white solid with a m.p. of 82–83 °C. ^1^H-NMR (400 MHz, CDCl_3_): δ_H_ 7.39 (d, *J* = 3.2 Hz, 1H, H-3), 7.09 (dd, *J* = 8.9, 3.2 Hz, 1H, H-5), 6,84 (d, *J* = 8.9 Hz, 1H, H-6), 5.06 (t, *J* = 7.2 Hz, 1H, H-6′), 2.78 (d, *J* = 16.7 Hz, 1H, H-2′), 2.66 (d, *J* = 16.7 Hz, 1H, H-2′), 2.12–2.08 (q, 2H, H-5′), 1.83–1.76 (m, 1H, H-4′), 1.67 (s, 3H, H-8′), 1.57 (s, 3H, H-9′), and 1.40 (s, 3H, H-10′). APT (100 MHz, CDCl_3_): δ_C_ 194.0 (C-1′), 154.2 (C-1), 150.1 (C-4), 132.3 (C-7′), 125.4 (C-5), 123.3 (C-6′), 120.2 (C-2), 119.6 (C-6), 110.6 (C-3), 80.8 (C-3′), 47.4 (C-2′), 39.0 (C-4′), 25.6 (C-8′), 23.8 (C-10′), 22.2 (C-5′), and 17.6 (C-9′). HRMS (ESI) calc. for C_16_H_22_O_4_ [M + H]^+^: 278.1518, found: 278.1462. The spectroscopic data can be consulted in Figures S1–S5 in Supplementary Materials.

Debromocymopolone (**2**): An orange solid with a m.p. of 74–75 °C. ^1^H-NMR (400 MHz, CDCl_3_): δ_H_ 12.41 (s, 1H, OH), 7.25 (d, *J* = 3.0 Hz, 1H, H-3), 7.00 (dd, *J* = 8.9, 3.0 Hz, 1H, H-5), 6.88 (d, *J* = 8.9 Hz, 1H, H-6), 6.68 (s, 1H, H-2′), 5.35 (s, 1H, OH), 5.13 (t, *J* = 7.0 Hz, 1H, H-6′), 2.30–2.24 (m, 4H, H-5′y H-4′), 2.20 (s, 3H, H-10′), 1.73 (s, 3H, H-8′), and 1.64 (s, 3H, H-9′). APT (100 MHz, CDCl_3_): δ_C_ 196.9 (C-1′), 161.9 (C-3′), 157.9 (C-1), 148.1 (C-4), 133.6 (C-7′), 124.9 (C-5), 123.6 (C-6′), 121.2 (C-2), 120.3 (C-2′), 119.8 (C-6), 115.7 (C-3), 42.3 (C-4′), 26.9 (C-5′), 26.2 (C-10′), 20.8 (C-8′), and 18.8 (C-9′). The spectroscopic data were consistent with those reported in the literature for debromocypolone [42,43,44]. The spectroscopic data can be consulted in Figures S6 and S7 in Supplementary Materials.

1,4-dihydroxy-2-(3′,7′-dimethyl-1′-oxo-2′-*Z*-6′-octadienyl)-benzene (**3**): A yellow liquid. ^1^H- NMR (400 MHz, CDCl_3_): δ_H_ 12.50 (s, 1H, OH), 7.26 (d, *J* = 3.0 Hz, 1H, H-3), 7.02 (dd, *J* = 8.8, 3.0 Hz, 1H, H-5), 6.88 (d, *J* = 8.8 Hz, 1H, H-6), 6.65 (s, 1H, H-2′), 5.14 (t, *J* = 7.6 Hz, 1H, H-6′), 2.62 (t, *J* = 7.6 Hz, 2H, H-4′), 2.22 (q, *J* = 7.5 Hz, 2H, H-5′), 2.01 (s, 3H, H-10′), 1.65 (s, 3H, H-8′), and 1.63 (s, 3H, H-9′). APT (100 MHz, CDCl_3_): δ_C_ 195.5 (C-1′), 161.5 (C-3′), 156.9 (C-1), 147.2 (C-4), 132.4 (C-7′), 124.1 (C-5), 123.3 (C-6′), 120.3 (C-2), 120.0 (C-2′), 118.9 (C-6), 114.9 (C-3), 34.3 (C-4′), 26.6 (C-5′), 25.8 (C-8′), 25.4 (C-10′), and 17.5 (C-9′). The spectroscopic data were consistent with those reported in the literature [42,43,44]. The spectroscopic data can be consulted in Figures S8 and S9 in Supplementary Materials.

lhotzcromene (**4**): A white solid with a m.p. of 48–49 °C. ^1^H-NMR (400 MHz, CDCl_3_): δ_H_ 11.7 (s, 1H, OH), 7.89 (dd, *J* = 8.5, 2.2 Hz, 1H, H-7), 7.76 (d, *J* = 2.2 Hz, 1H, H-5), 6.82 (d, *J* = 8.5 Hz, 1H, H-8), 6.42 (d, *J* = 10.0 Hz, 1H, H-4), 5.63 (d, *J* = 10.0 Hz, 1H, H-3), 5.10 (t, *J* = 5.6, 1.4 Hz, 1H, H-3′), 2.14–2.11 (m, 2H, H-2′), 1.84–1.76 (m, 1H, H-1′), 1.72–1.65 (m, 1H, H-1′), 1.67 (s, 3H, H-1″),1.58 (s, 3H, H-5′), and 1.44 (s, 3H, H-6′). APT (100 MHz, CDCl_3_): δ_C_ 172.3 (C-9), 158.4 (C-8a), 131.9 (C-7), 131.8 (C-4′), 129.9 (C-3), 128.8 (C-5), 123.8 (C-3′), 122.2 (C-4), 121.4 (C-6), 120.5 (C-4a), 116.0 (C-8), 80.0 (C-2), 41.7 (C-1′), 27.1 (C-1″), 25.6 (C-5′), 22.7 (C-2′), and 17.6 (C-6′). The spectroscopic data were consistent with those reported in the literature for lhotzchromene [47]. The spectroscopic data can be consulted in Figures S11 and S12 in Supplementary Materials.

Ceanothifoliol (**5**): A yellow solid with a m.p. of 64–66 °C. ^1^H-NMR (400 MHz, acetone-d_6_): δ_H_ 11.75 (s, 1H, OH), 8.14 (s, 1H, OH), 7.31 (d, *J* = 3.0 Hz, 1H, H-3), 7.11 (dd, *J* = 8.8, 3.0 Hz, 1H, H-5), 6.82 (d, *J* = 8.8 Hz, 1H, H-6), 2.86 (s, 3H, H-2′), and 2.63 (s, 3H, H-1″). APT (100 MHz, acetone-d_6_): δ_C_ 155.6 (C-1), 149.3 (C-4), 124.8 (C-2), 119.4 (C-3), 118.5 (C-6), 115.5 (C-5), 88.6 (C-1′), 29.6 (C-2′), and 26.0 (C-1″). The spectroscopic data can be consulted in Figures S13 and S14 in Supplementary Materials.

### 3.5. Preparation of Ceanothichromone **6** Starting from **2**

Compound **2** was subjected to an intramolecular cyclization reaction of the oxo-Michael type, adapting the methodology described in the literature for similar compounds [43]. In a typical experiment, 100.0 mg of **2** (0.100 mmol) and 10.0 mL of 10% NaOH were added to a round-bottomed flask. The resulting mixture was continuously stirred at room temperature for a period of approximately 12 h. The reaction was monitored by TLC to verify the disappearance of compound **2**. Subsequently, 10% HCl was added slowly until the mixture was neutralized and then a liquid–liquid extraction was carried out with AcOEt (3 × 25 mL). The organic phases were combined, dried over anhydrous Na_2_SO_4_, and the solvent was distilled off under reduced pressure. In this way, a brown oily liquid corresponding to compound **6** (92.0 mg, 92.0%) was obtained.

**Ceanothichromone** (**6**): A brown oily liquid. ^1^H-NMR (400 MHz, CDCl_3_): δ_H_ 2.80 (d, *J* = 16.7 Hz, 1H, H-3), 7.38 (s, 1H, H-5), 7.09 (dd, *J* = 9.1, 3.1 Hz, 1H, H-7), and 6.87 (d, *J* = 9.1 Hz, 1H, H-8). APT (100 MHz, CDCl_3_): δ_C_ 194.8 (CO), 157.1 (C-8a), 154.5 (C-6), 125.0 (C-7), 120.3 (C-4a), 119.6 (C-3′), 110.7 (C-8), 80.8 (C-2), and 47.5 (C-3). The spectroscopic data can be consulted in Figure S15 and Figure S19 in Supplementary Materials.

### 3.6. Antifungal Potential of Chemical Constituents of P. ceanothifolium

#### 3.6.1. Inhibition of Mycelial Growth Assay

The antifungal potential against *M. roreri*, *F. solani*, and *L. theobromae* of the compounds was determined by the inhibition of mycelial growth assay, adapting the poisoned food technique with some modifications [50]. Stock solutions of the compounds were prepared with concentrations between 2000 μg/mL and 62.5 μg/mL in EtOH and then these were mixed with the potato dextrose agar (PDA) medium to obtain final concentrations per well of 100 μg/mL, 50 μg/mL, 25 μg/mL, 12.5 μg/mL, 6.5 μg/mL, and 3.2 μg/mL. In the center of each well, a 2 mm-diameter plug of fungus was inoculated and the boxes were incubated at 25 °C (15 days for *M. roreri*, 3 days for *F. solani*, and 8 days for *L. theobromae*). 2% ethanol was used as the negative control, PDA medium was used as the blank, and benomyl and mancozeb were used as the positive controls. After the incubation time, the plates were scanned and the mycelial growth area in each well was determined using the IMAGE J image processing program. The data obtained were used to determine the mycelial growth inhibition percentage (% ICM) using Equation (1), which is as follows:

% ICM = [(C − T)/C] × 100 (1)

where C is the radial growth of the fungus in the blank and T the radial growth of the fungus with the treatment.

With the % ICM and the concentrations evaluated, the IC_50_ were determined by means of a non-linear regression analysis using the GraphPad Prism 8 program. All the reported results correspond to the average of nine independent replicates (n = 9), together with its 95% confidence interval.

#### 3.6.2. Determination of the Fungicidal or Fungistatic Effect

The fungicidal or fungistatic effect was determined for the active compounds following the methodology reported in the literature with some modifications [51]. The plug of the fungus used in each of the wells where no apparent growth was observed when evaluating a concentration of 100 µg/mL of each compound in the mycelial growth inhibition assay was inoculated into sterile PDA medium. It was incubated at 25 °C for 15 days for *M. roreri*, 8 days for *L. theobromae*, and 5 days for *F. solani*. A fungistatic effect was defined as the development of a fungal colony from the inoculum, while the absence of colony growth was a fungicidal effect. For each compound, three independent tests were performed, each with nine independent replicates (n = 9). Benomyl and mancozeb were used as positive controls.

#### 3.6.3. Inhibition of conidial germination

The percentage of inhibition of conidial germination for the active compounds for which the IC_50_ was determined was done following the methodology reported in the literature with some modifications [52]. The method involved mixing a test tube of culture medium (glucose-yeast extract for *L. theobromae* and agar-water for *F. solani* and *M. roreri*) with the solution of the compound to be evaluated, ensuring that the concentration end of the compound was the respective IC_50_. The mixture was allowed to solidify in a Petri dish, then 10 μL of a conidial suspension (105 conidia/mL) was applied and a coverslip was placed on top. Each assembly was incubated at 35 °C and the conidial germination reading was performed after 7 h for *F. solani* and 72 h for *L. theobromae* and *M. roreri*. For the conidial germination reading, 20 conidia were counted in 5 different fields, for a total of 100 conidia, and it was determined that the germinated conidia were those that presented twice the length of the germ tube with respect to the length of the same conidium. The percentage of inhibition of conidia germination (% ICG) was determined with Equation (2), which is as follows:

% ICG = [(B − T)/B] × 100 (2)

where B is the conidia germinated in the control (without treatment) and T is the conidia germinated in the treatment.

Benomyl and mancozeb were used as positive controls, evaluated at their respective IC_50_. For each trial, three independent trials were performed, each with nine independent replicates (n = 9). The % ICGs were calculated, which are reported together with their standard deviations.

### 3.7. Data Analysis

The one-way ANOVA statistical test was performed considering the assumptions of the test (normality, homogeneity of variances, independence, randomness, and outliers) to determine if there were significant differences in the trials. The data that presented significant differences were subjected to additional multiple comparison tests, such as a Dunnet for normal data, to confirm in which group the differences occurred. These analyses were performed in the Graphpad Prism 8 statistical program. All the results reported correspond to the mean of nine independent replicates (n = 9) and their respective standard deviation, using a statistical significance of *p* < 0.05, *p* < 0.005, *p* < 0.001, and *p* < 0.0001.

## 4. Conclusions

This study corresponds to the first report on the chemical composition and antifungal activity of substances from *P. ceanothifolium*, highlighting the first report in the literature for ceanothifolione **1**, ceanothifoliol **5**, and ceanothichromone **6**. In addition, this research provides the first evidence of fungicidal and fungistatic effects, and the inhibition of conidial germination for compounds **1**–**6** against three phytopathogens of importance in cocoa cultivation (*M. roreri*, *F. solani*, and *L. theobromae*). The strong antifungal activity observed, particularly for **1**, **2**, and **6**, indicates the potential of these bioactive compounds as promising biocontrol agents for the management of major diseases in cocoa crops.

## Figures and Tables

**Table 1:** Determination of the antifungal activity of the ethanolic extract and fractions from *P. ceanothifolium* inflorescences against *F. solani*, *L. theobromae*, and *M. roreri*.

Substance	*F. solani*		*L. theobromae*		*M. roreri*	
	^a^ % IMG	^b^ IC_50_ (ppm)	^a^ % IMG	^b^ IC_50_ (ppm)	^a^ % IMG	^b^ IC_50_ (ppm)
Extract	83.0 ± 3.8	198.8 (184.9–214.1)	92.0 ± 2.4	503.9 (449.6–561.7)	93.2 ± 0.8	129.5 (116.9–144.0)
DCM	100.0 ± 0.0	195.7 (180.4–217.9)	82.7 ± 2.5	396.6 (353.8–442.1)	100.0 ± 0.0	102.7 (92.7–114.2)
EtOAc	35.3 ± 2.5	N.D	19.0 ± 3.6	N.D	10.0 ± 3.5	N.D
IPA	12.3 ± 2.1	N.D	13.7 ± 2.1	N.D	5.0 ± 2.3	N.D
EtOH:H_2_O	3.3 ± 3.1	N.D	4.0 ± 1.0	N.D	0.0 ± 0.0	N.D
Benomyl	100.0 ± 0.0	3.7 (2.7–5.1)	100.0 ± 0.0	5.2 (4.2–6.3)	100.0 ± 0.0	15.0 (13.9–16.1)
Mancozeb	100.0 ± 0.0	5.3 (4.7–5.9)	100.0 ± 0.0	5.9 (5.2–6.9)	100.0 ± 0.0	3.4 (3.0–3.6)

a Percentage of inhibition of mycelial growth (% IMG) results are determined at the 1000 µg/mL concentration. b Inhibitory concentration 50 (IC_50_) expressed as the mean of nine independent replicates (n = 9), along with 95% confidence intervals. N.D = Not determined.

**Table 2:** Inhibitory capacity on mycelial growth of chemical constituents from *P. ceanothifolium*.

Compound	*F. solani*	*L. theobromae*	*M. roreri*
	^a^ IC_50_ μM	^a^ IC_50_ μMa	^a^ IC_50_ μM
1	93.8 ± 2.1 ****	30.4 ± 2.1 *	112.7 ± 2.6 ***
2	34.2 ± 2.2 *	63.2 ± 2.1 *	60.0 ± 0.9 *
3	68.8 ± 2.4 **	110.9 ± 2.2 **	92.3 ± 5.6 **
4	29.6 ± 2.2 *	67.0 ± 2.1 *	199.2 ± 2.3 ****
5	ND	ND	ND
6	16.9 ± 2.2 *	81.4 ± 2.1 **	186.0 ± 2.8 ****
Mancozeb	9.7 ± 2.1	10.8 ± 1.1	6.3 ± 0.3

a Expressed as ± SD (standard deviation), where n = 9. *p*-value represent statistically significant differences * (*p* < 0.05), ** (*p* < 0.005), *** (*p* < 0.001), **** (*p* < 0.0001) (Dunnett ’s test); and N.D.: not determined.

**Table 3:** Percentages of inhibition of conidial germination (% ICG) of *Piper* derived compounds with antifungal activity against *M. roreri*, *F. solani*, and *L. theobromae*.

Compound/Fungal	*F. solani*	*L. theobromae*	*M. roreri*
	^a^ % ICG	^a^ % ICG	^a^ % ICG
1	46.3 ± 1.9 *	40.9 ± 1.2 *	73.0 ± 3.4 *
2	32.7 ± 2.1 *	45.9 ± 1.2 *	81.2 ± 2.7 *
3	25.5 ± 1.7 *	65.4 ± 2.3 *	68.4 ± 5.2 *
4	55.2 ± 2.0 *	35.2 ± 1.7 *	60.3 ± 5.0 *
6	85.2 ± 1.5 *	75.5 ± 1.0 *	66.7 ± 4.3 *
Mancozeb	96.5 ± 2.1	95.5 ± 1.4	91.7 ± 2.5

a Evaluation at IC_50._ Expressed as ± SD (standard deviation), where n = 9. *p*-value represent statistically significant differences * (*p* < 0.0001) (Dunnett ’s test).

## Data Availability

Data are contained within the article and Supplementary Materials.

## Supplementary Materials

The following supporting information can be downloaded at: https://www.mdpi.com/article/10.3390/plants14060934/s1, The Supplementary Materials are available online and correspond to the isolation scheme of compounds **1**–**5** from *P. ceanothifolium* (Scheme S1). 1D and D NMR Spectra of compounds **1** to **6** (Figures S1–S19). Finally, Strains of phytopathogenic fungi used in the bioassays: and the results of the fungicide and fungistatic assay of the compounds with the greatest porcentage inhibition of mycelial growth of phytopathogenic associated of cocoa (Figures S20 and S21).
