# Supplementary material for: Chemical Constituents and Antifungal Properties of Piper ceanothifolium Kunth Against Phytopathogens Associated with Cocoa Crops
Source: Plants (Basel). 2025 Mar 16;14(6):934. doi: 10.3390/plants14060934 (PMC11945235; doi:10.3390/plants14060934)
Supplement: Supplementary file 1 [file plants-14-00934-s001.zip › plants-3465600-supplementary.pdf]

plants

MDPI

Supplementary materials

Table of content

1. Phytochemical study carried out on inflorescences from *Piper ceanothifolium.*

Scheme S1. Isolation scheme of compounds from *Piper ceanothifolium*

2. NMR spectra from phytochemistry isolation

Figure S1. ^{1}H-NMR spectra of compound **1** (400 MHz, $CDCl_3$).
Figure S2. APT spectra of compound **1** (100 MHz, $CDCl_3$).
Figure S3. COSY spectra of compound **1** ($CDCl_3$).
Figure S4. HMQC spectra of compound **1** ($CDCl_3$).
Figure S5. HMBC spectra of compound **1** ($CDCl_3$).
Figure S6. ^{1}H-NMR spectra of compound **2** (400 MHz, $CDCl_3$).
Figure S7. APT spectra of compound **2** (100 MHz, $CDCl_3$).
Figure S8. ^{1}H-NMR spectra of compound **3** (400 MHz, $CDCl_3$).
Figure S9. APT spectra of compound **3** (100 MHz, $CDCl_3$).
Figure S10. NOESY-1d spectra and correlations of compounds **2** and **3**.
Figure S11. ^{1}H-NMR spectra of compound **4** (400 MHz, $CDCl_3$).
Figure S12. APT spectra of compound **4** (100 MHz, $CDCl_3$).
Figure S13. ^{1}H-NMR spectra of compound **5** (400 MHz, $(CD_3)_2CO$).
Figure S14. APT spectra of compound **5** (400 MHz, $(CD_3)_2CO$).
Figure S15. ^{1}H-NMR spectra of compound **6** (400 MHz, $CDCl_3$).
Figure S16. APT spectra of compound **6** (100 MHz, $CDCl_3$).
Figure S17. COSY spectra of compound **6** ($CDCl_3$).
Figure S18. HMQC spectra of compound **6** ($CDCl_3$).
Figure S19. HMBC spectra of compound **6** ($CDCl_3$)

3. **Bioassays**

Figure S20. Fungicidal and fungistatic assay of the compounds with the greatest potential to inhibit the mycelial growth of **A**. *F. solani*, **B**. *M. roreri* and **C.** *L. theobromae*. Control (+): Blank of the IMG assay.

Figure S21. Strains of phytopathogenic fungi used in the bioassays: **A1)** *F. solani* strain in PDA; **A2)** Microscopy of *F. solani* on PDA; **B1)** *L. theobromae* strain in PDA; **B2)** Microscopy of *L. theobromae* on PDA; **C1)** *L. theobromae* strain on maltose-casamino acid agar medium**, C2)** *L. theobromae* strain on maltose-casamino acid agar medium; **C3)** Pycnidium of *L. theobromae***; D1)** *M. roreri* strain on PDA and **D2)** Microscopy of *M. roreri* on PDA. (Author's own).

1. **Phytochemical study carried out on inflorescences from *Piper ceanothifolium* Kunth.**

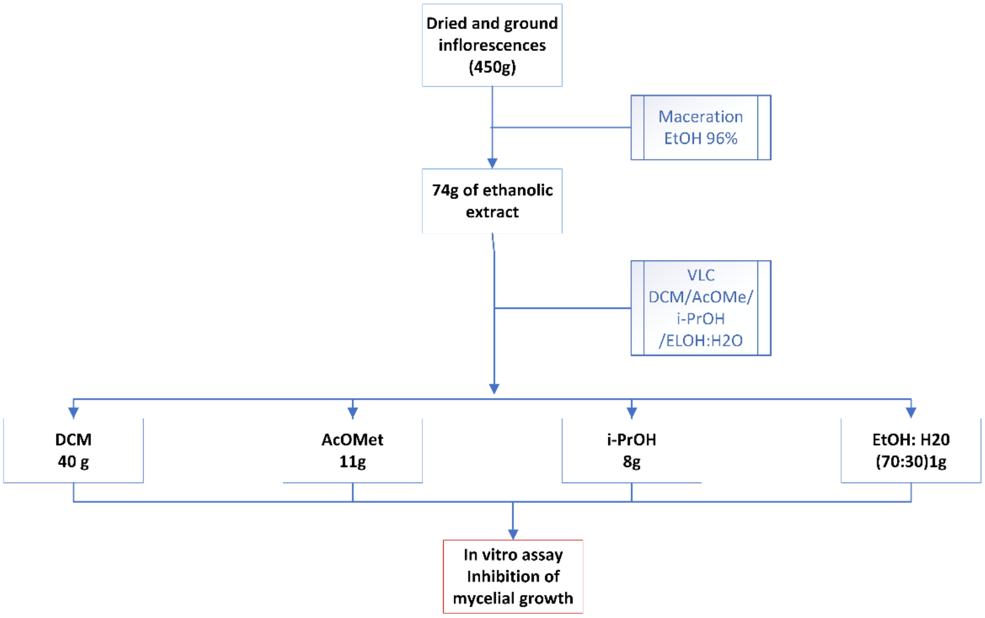

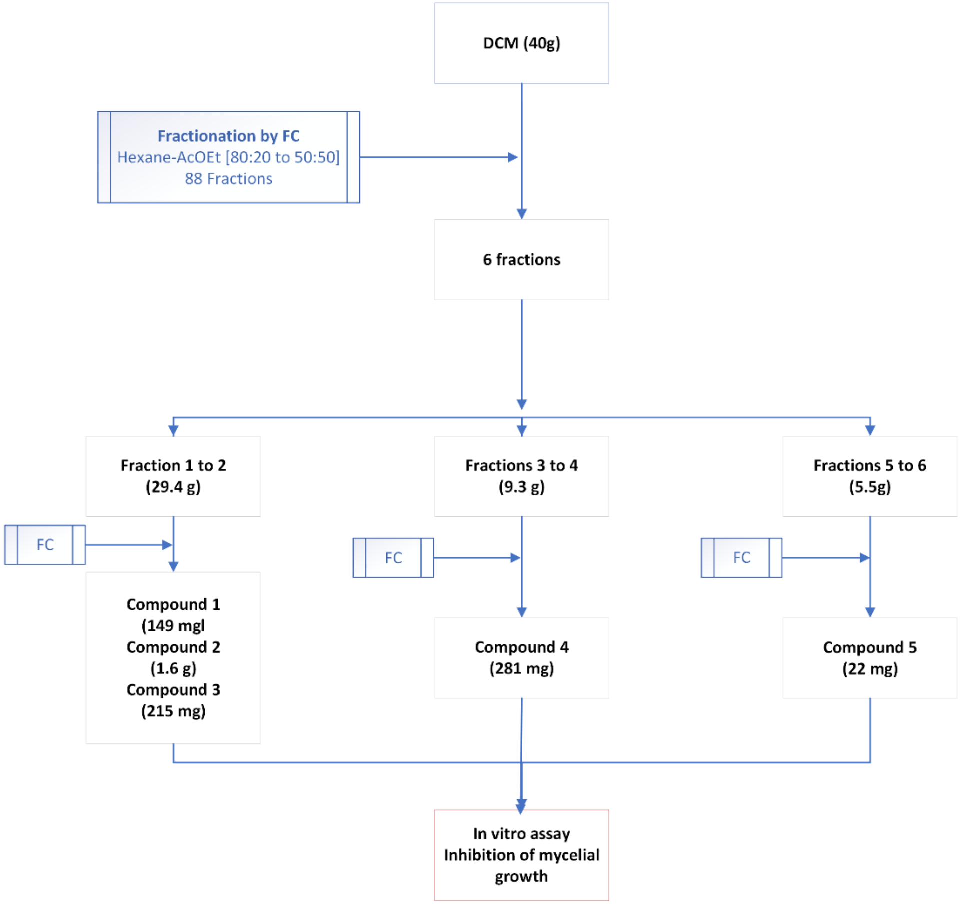

plants

MDPI

Scheme S1. Isolation scheme of compounds from *Piper ceanothifolium.*

2. NMR spectra from phytochemistry isolation

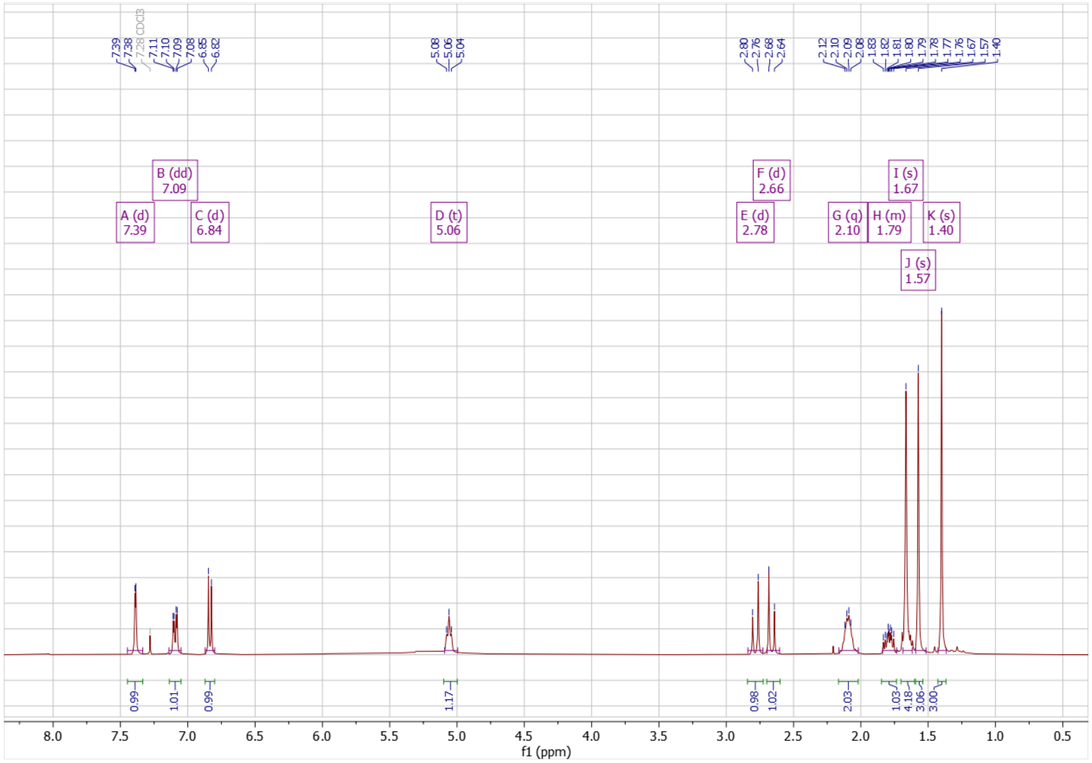

Figure S1. ^{1}H-NMR spectra of compound **1** (400 MHz, $CDCl_3$).

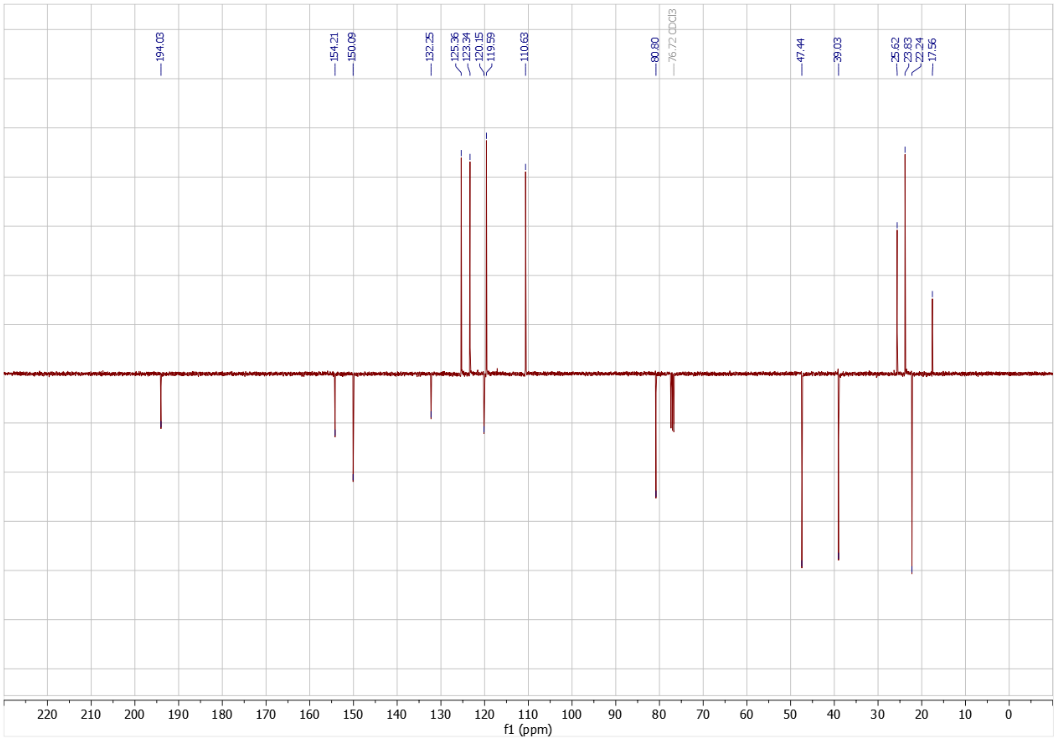

Figure S2. APT spectra of compound **1** (100 MHz, $CDCl_3$).

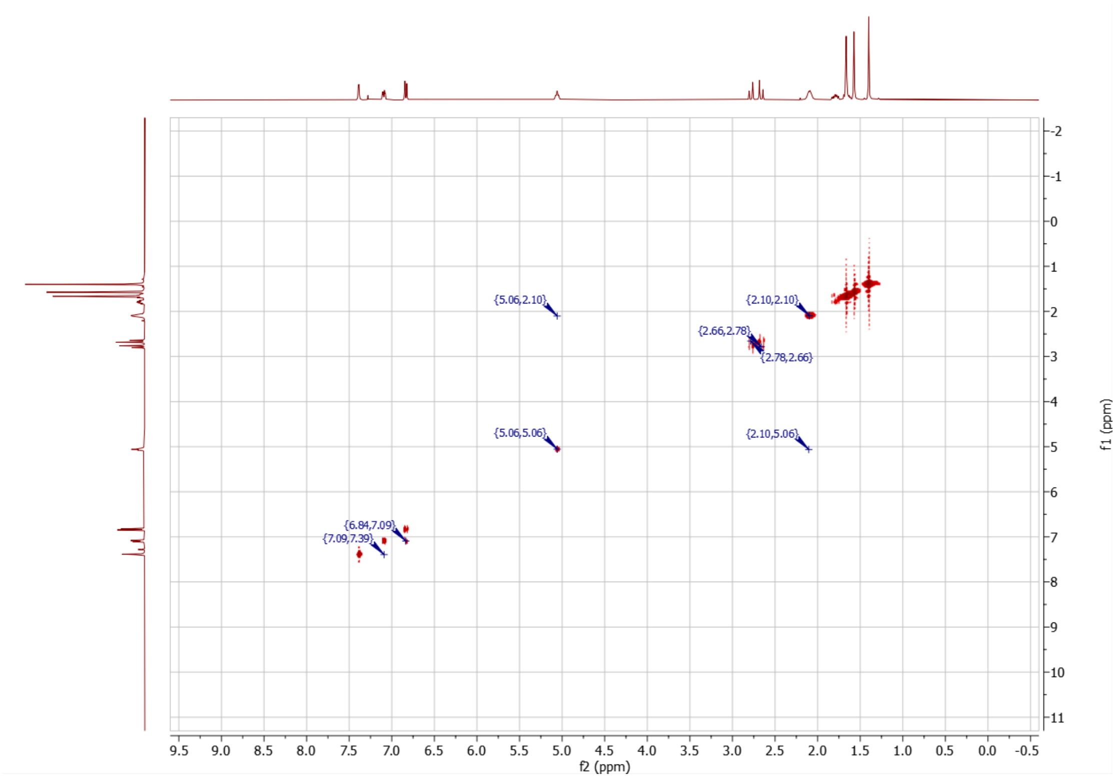

Figure S3. COSY spectra of compound **1** ($CDCl_3$).

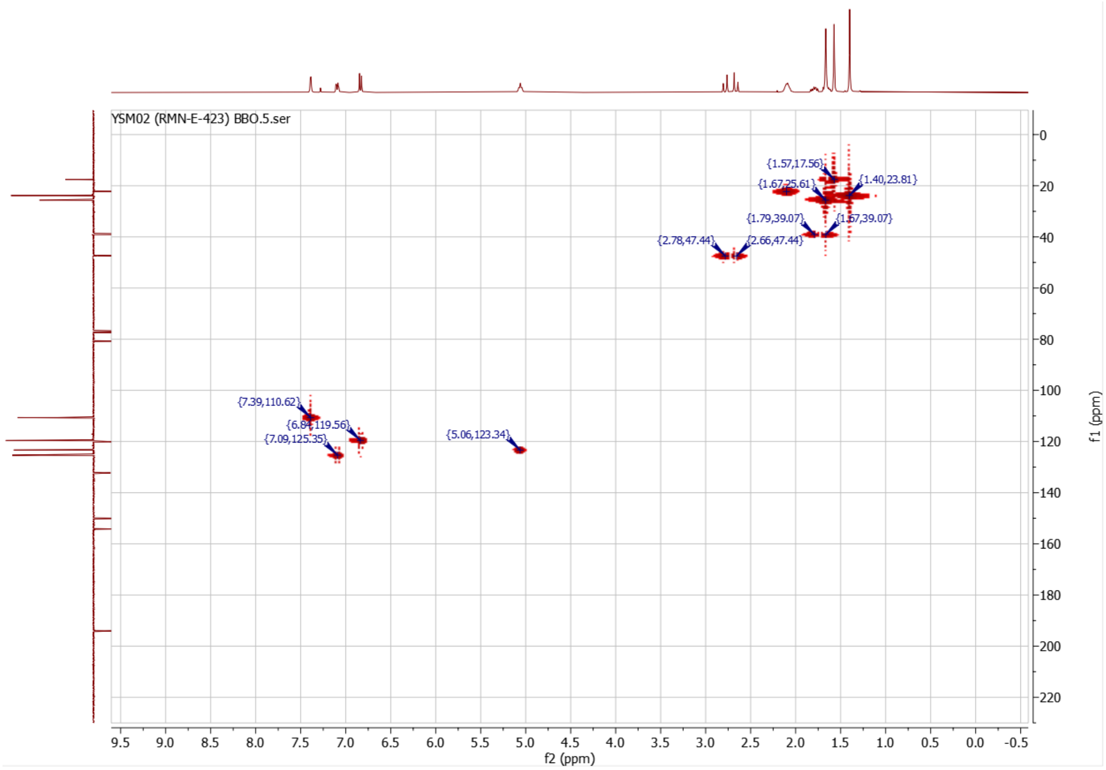

Figure S4. HMQC spectra of compound **1** ($CDCl_3$).

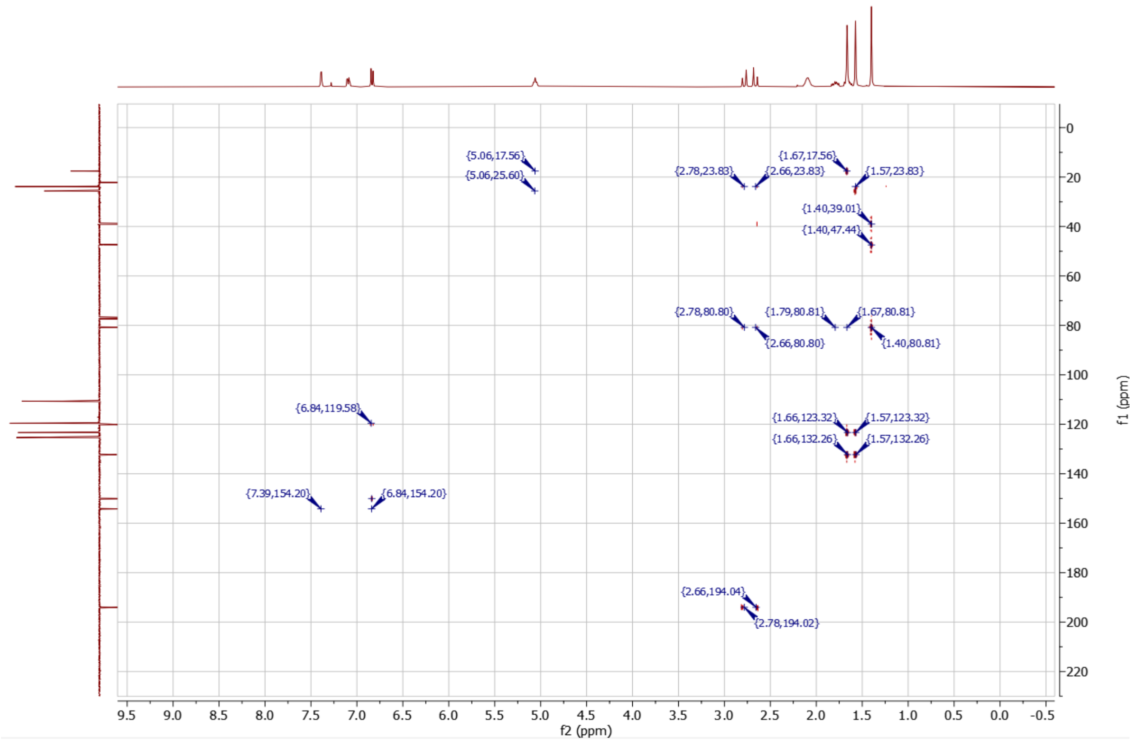

Figure S5. HMBC spectra of compound **1** ($CDCl_3$).

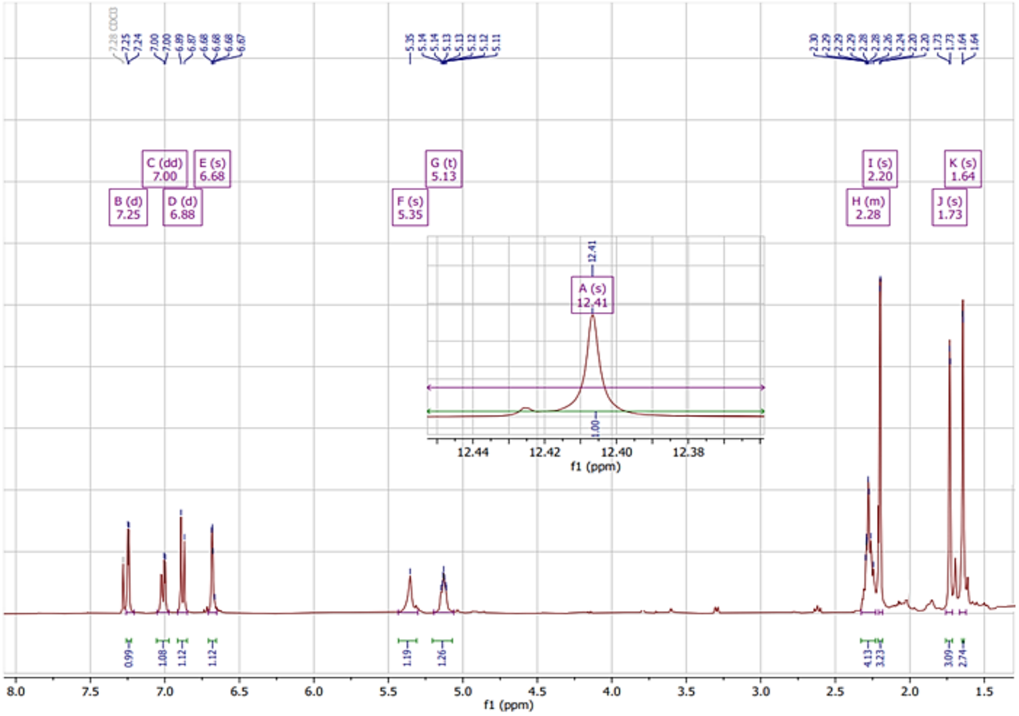

Figure S6. ^{1}H-NMR spectra of compound **2** (400 MHz, $CDCl_3$).

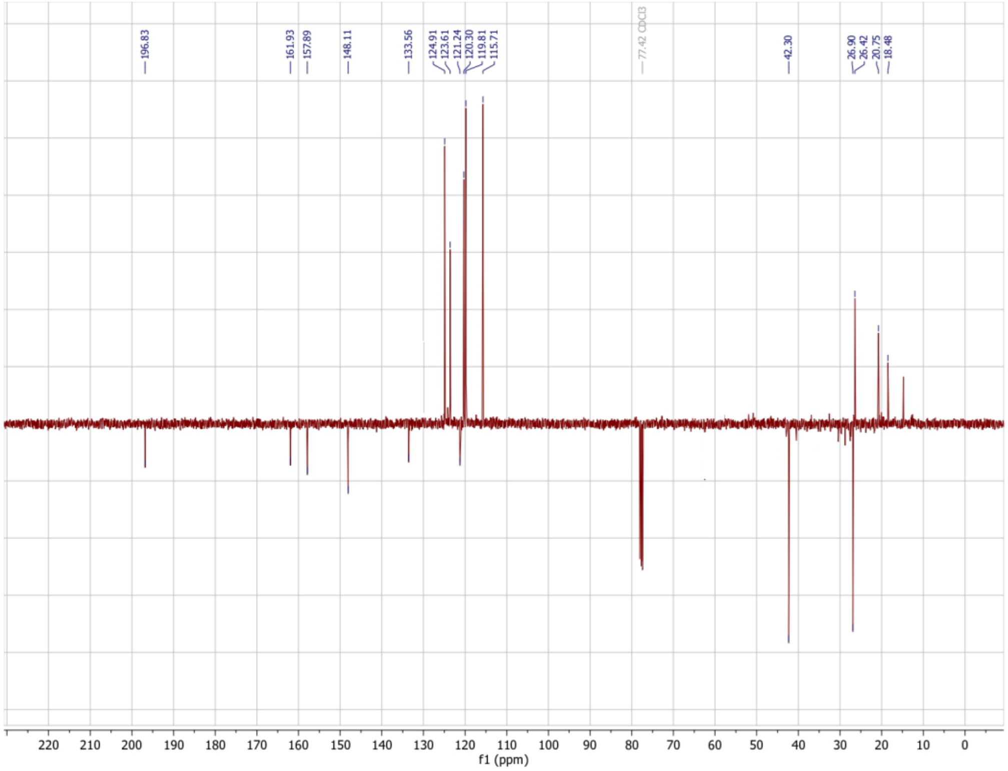

Figure S7. APT spectra of compound **2** (100 MHz, CDCl3).

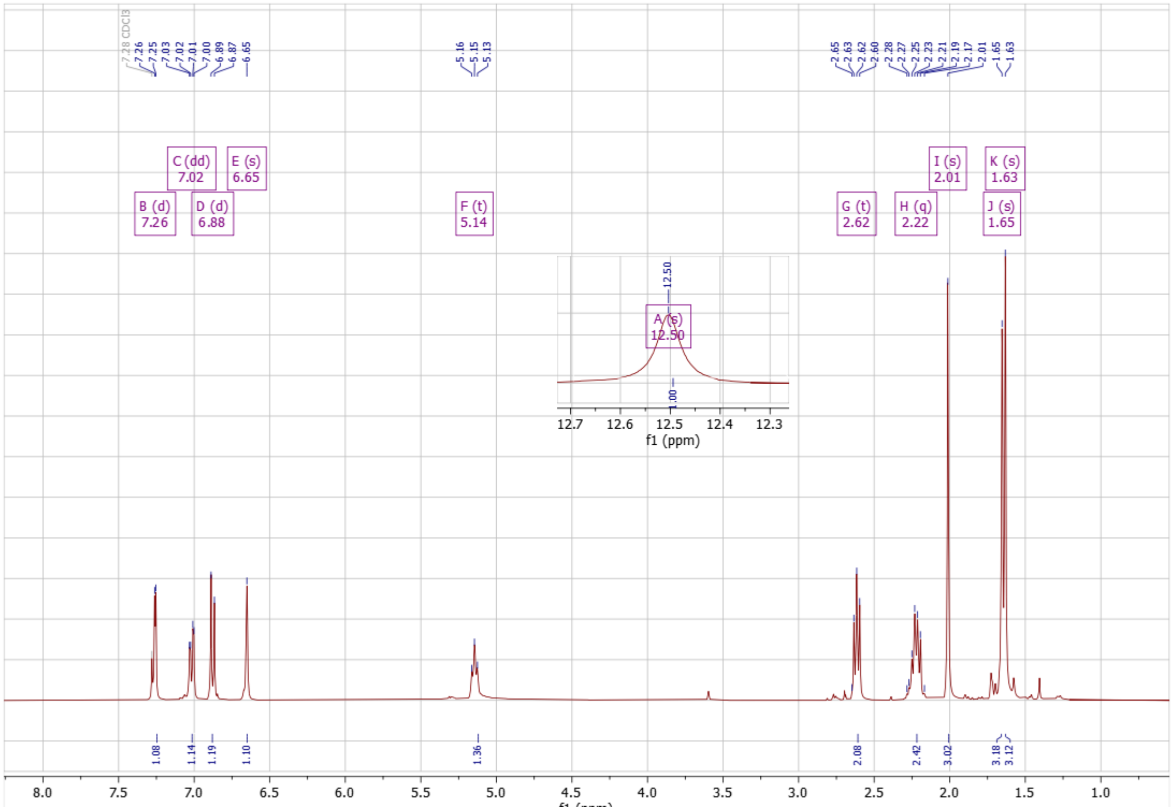

Figure S8. ^{1}H-NMR spectra of compound 3 (400 MHz, CDCl3).

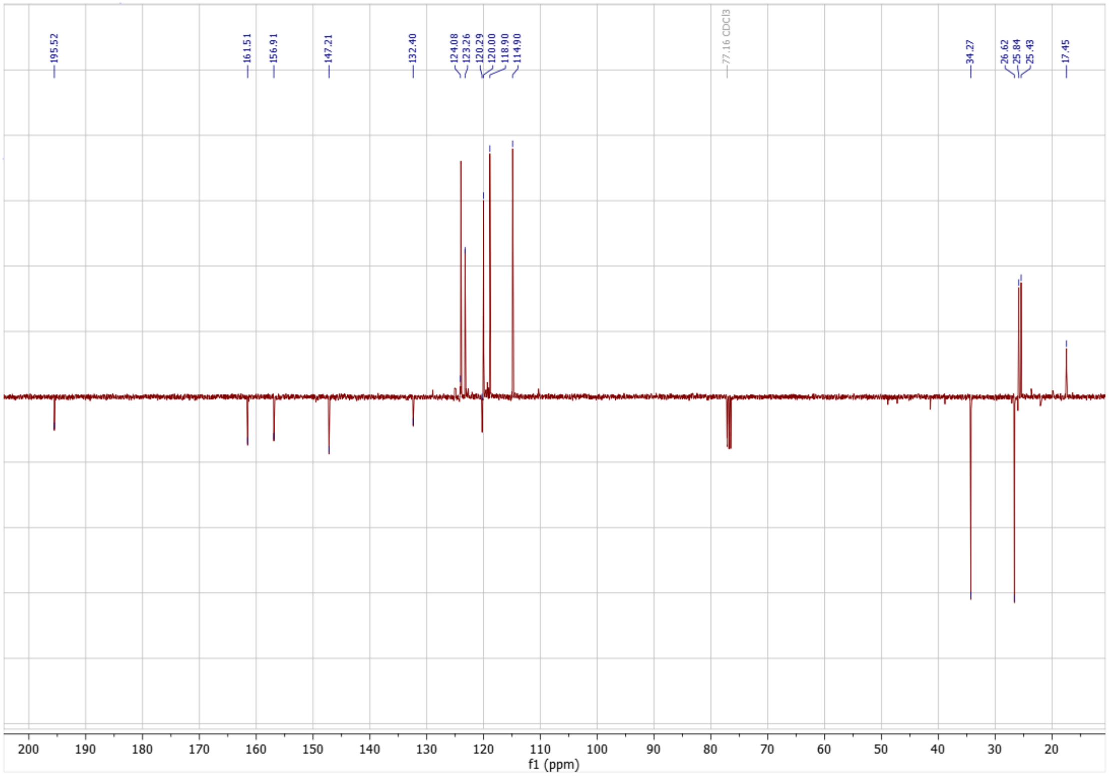

Figure S9. APT spectra of compound **3** (100 MHz, $CDCl_3$).

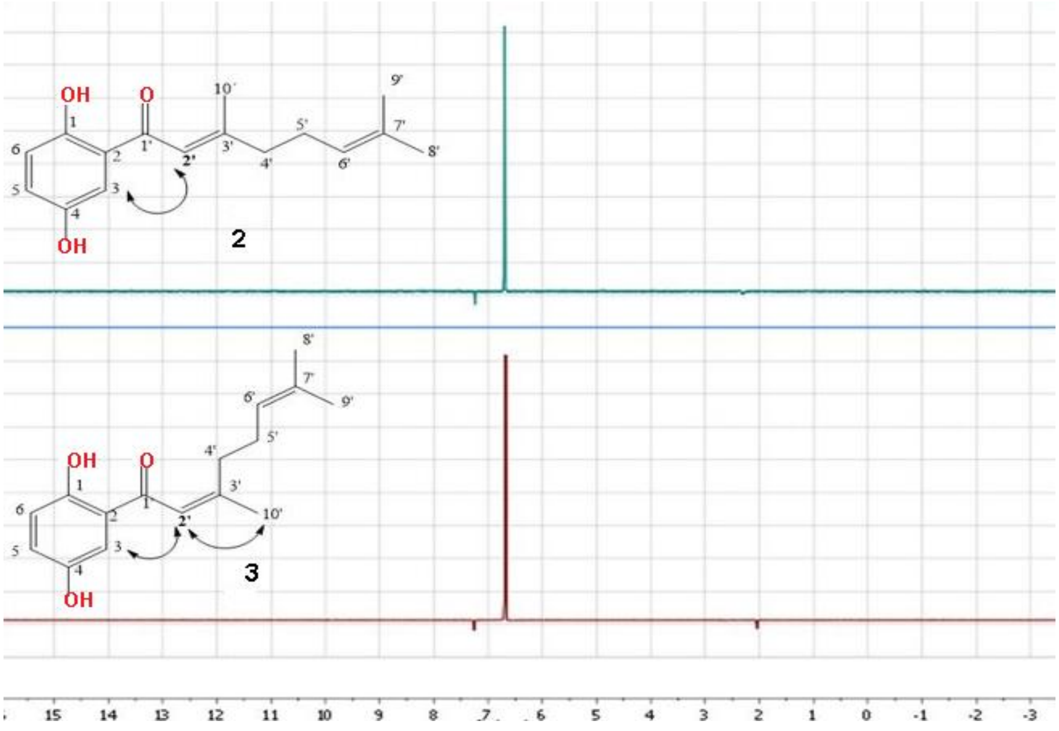

Figure S10. NOESY-1d spectra and correlations of compounds **2** and **3**.

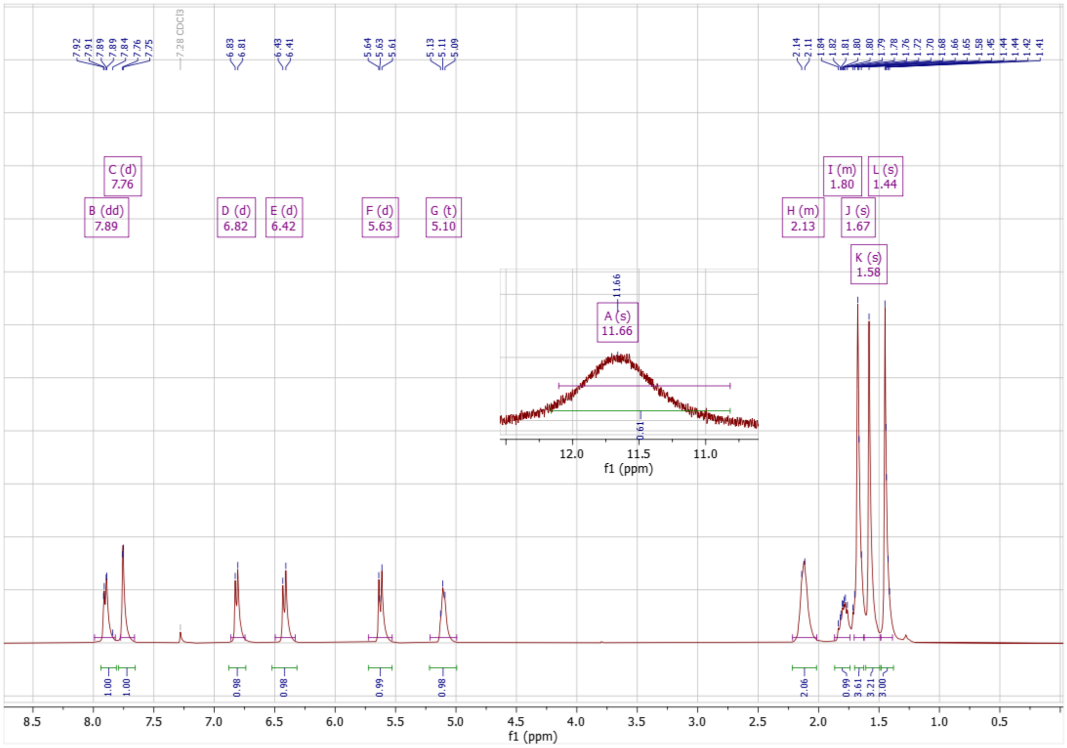

Figure S11. ^{1}H-NMR spectra of compound **4** (400 MHz, $CDCl_3$).

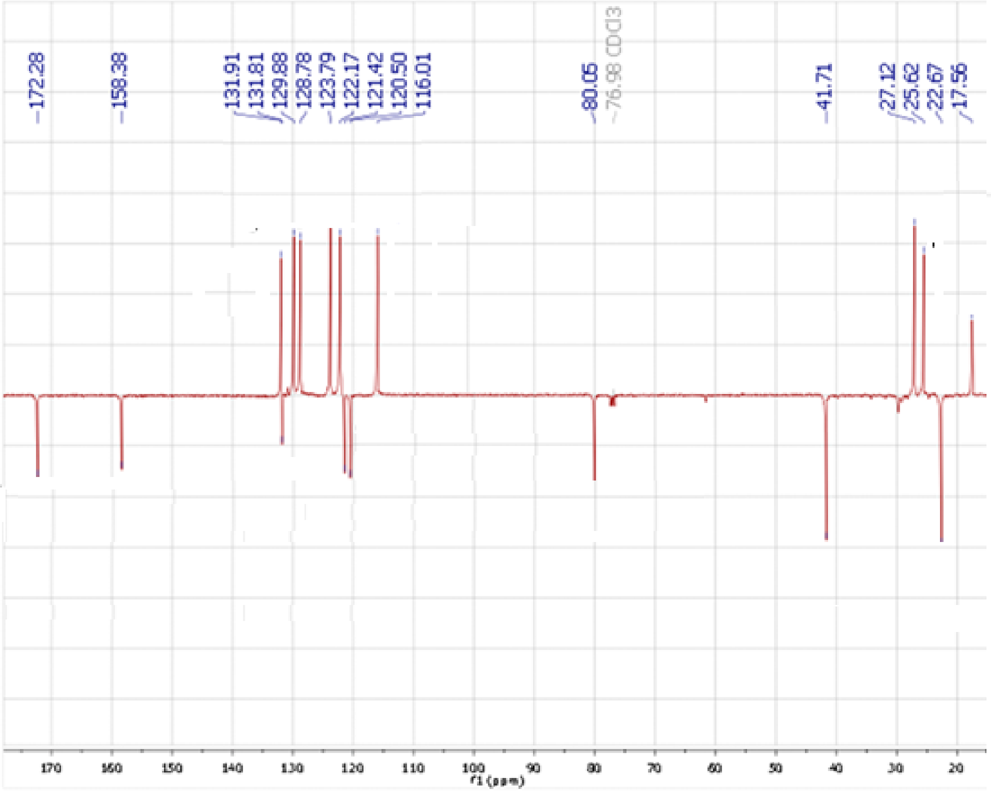

Figure S12. APT spectra of compound **4** (100 MHz, $CDCl_3$).

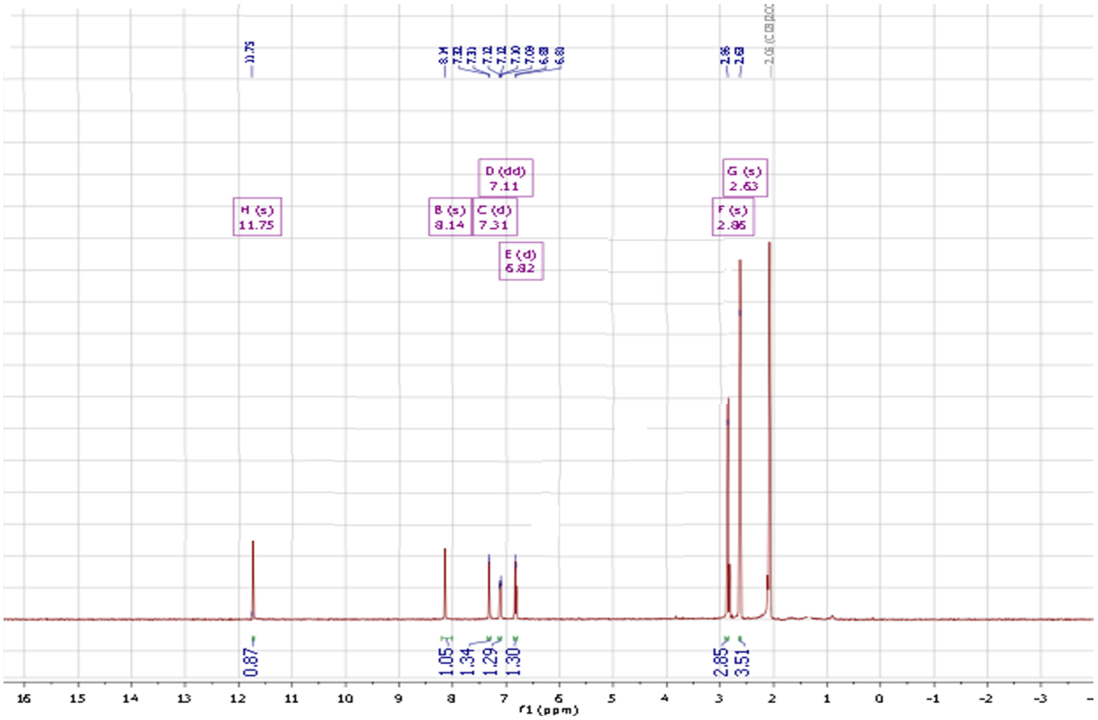

Figure S13. ^{1}H-NMR spectra of compound 5 (400 MHz, $(CD_3)_2CO$).

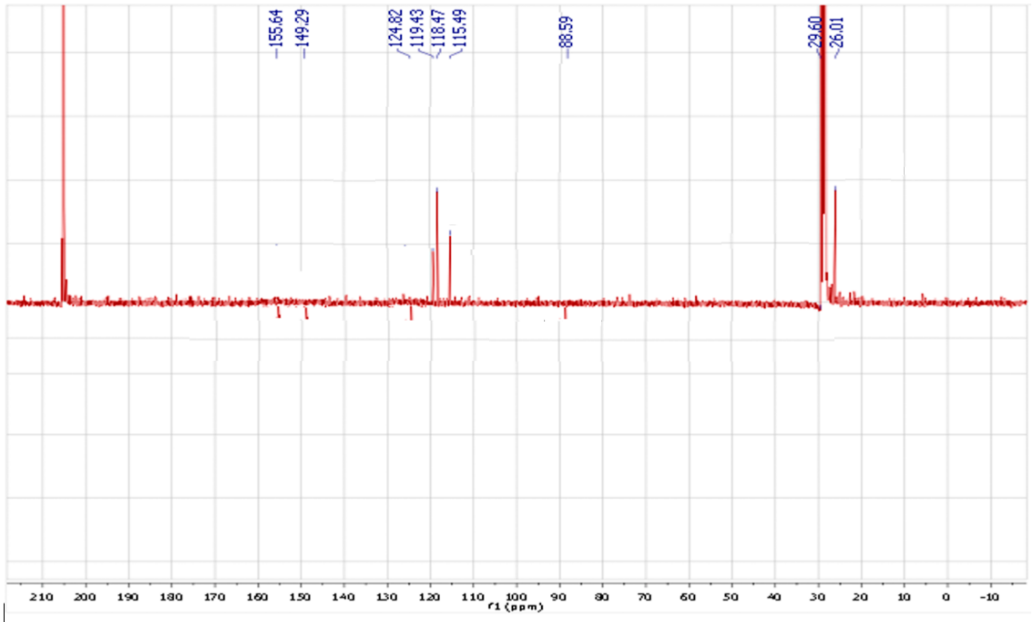

Figure S14. APT spectra of compound **5** (400 MHz, $(CD_3)_2CO$).

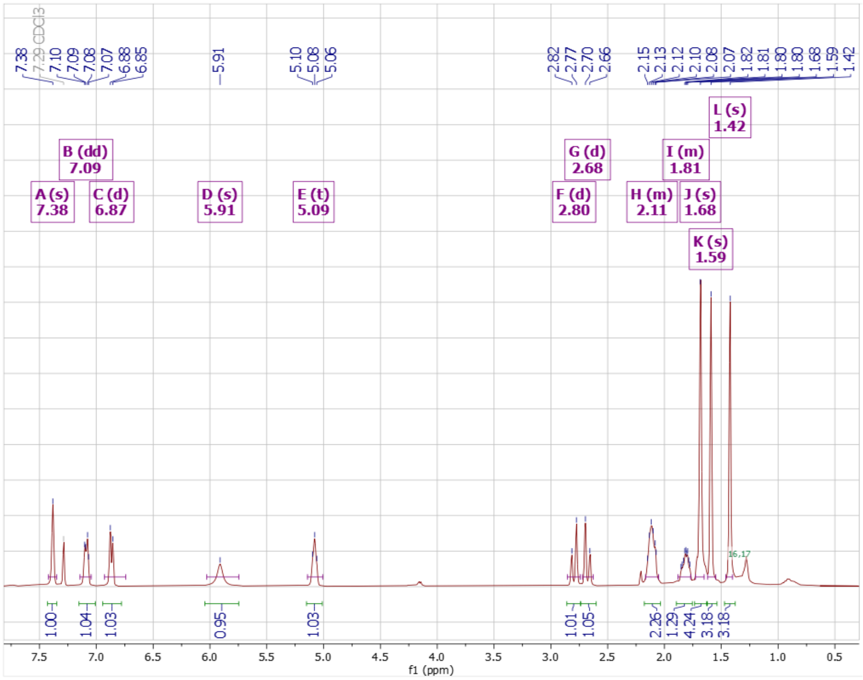

Figure S15. ^{1}H-NMR spectra of compound **6** (400 MHz, $CDCl_3$).

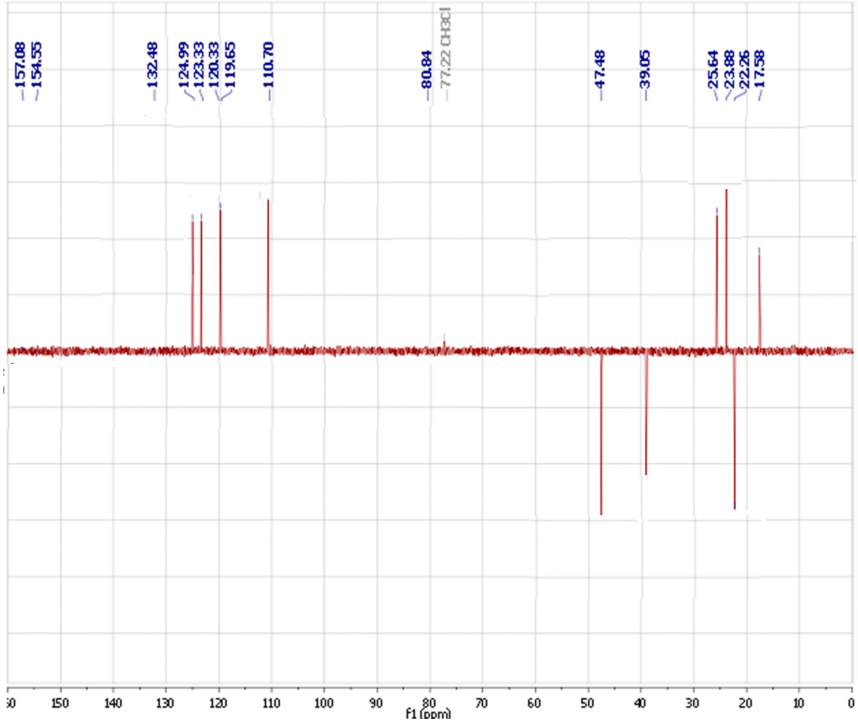

Figure S16. APT spectra of compound **6** (100 MHz, $CDCl_3$).

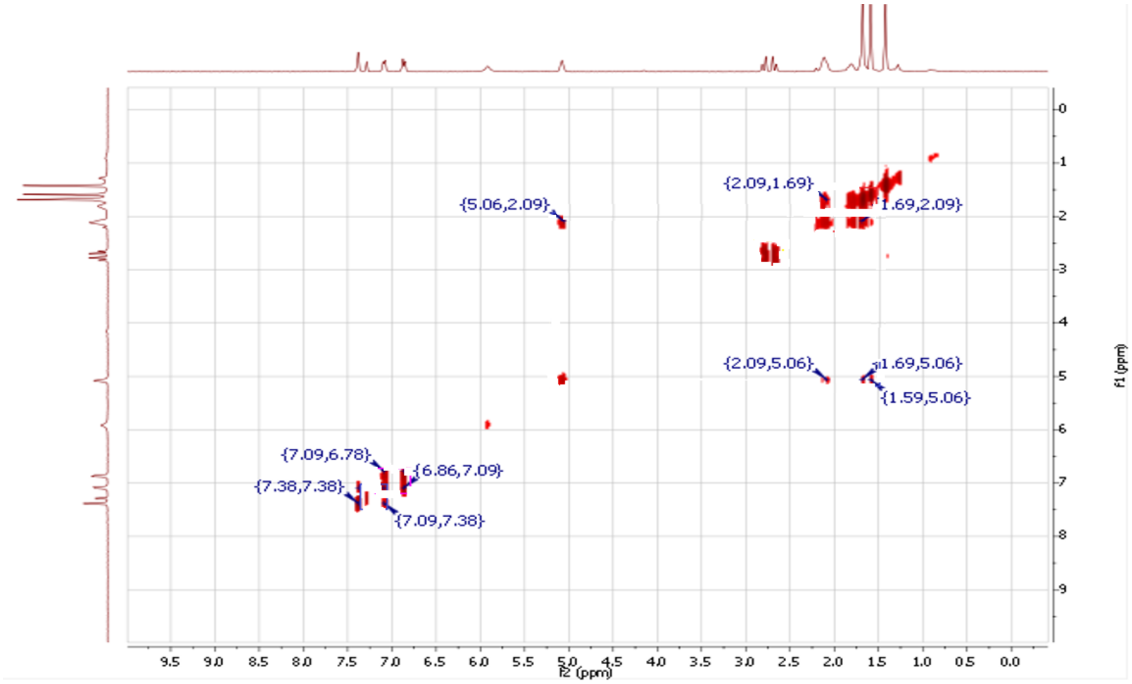

Figure S17. COSY spectra of compound **6** ($CDCl_3$).

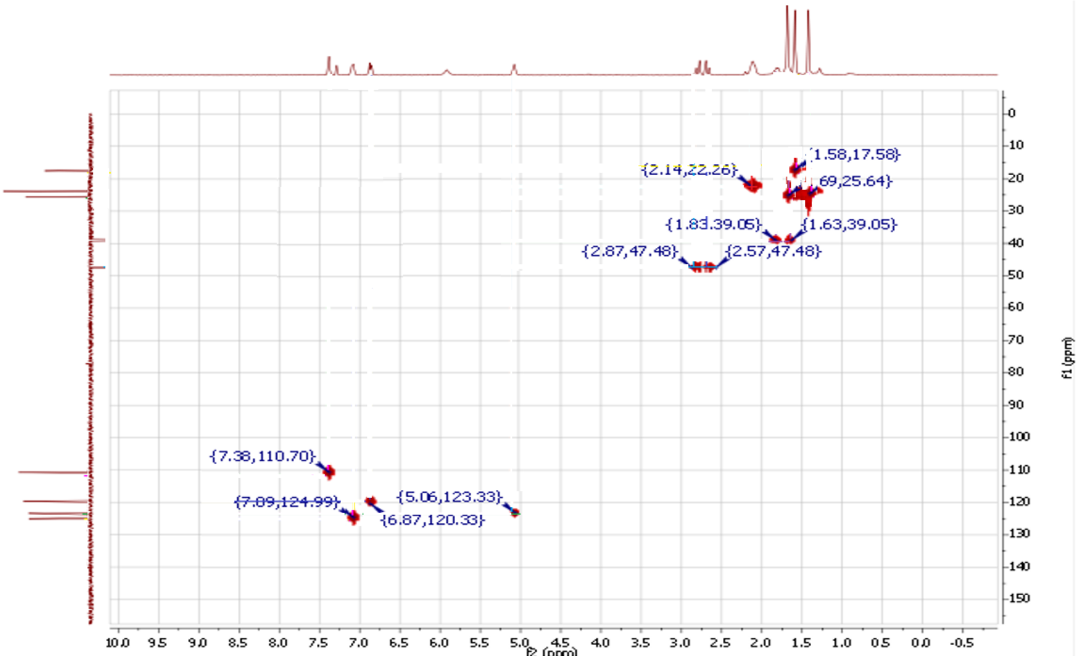

Figure S18. HMQC spectra of compound **6** ($CDCl_3$).

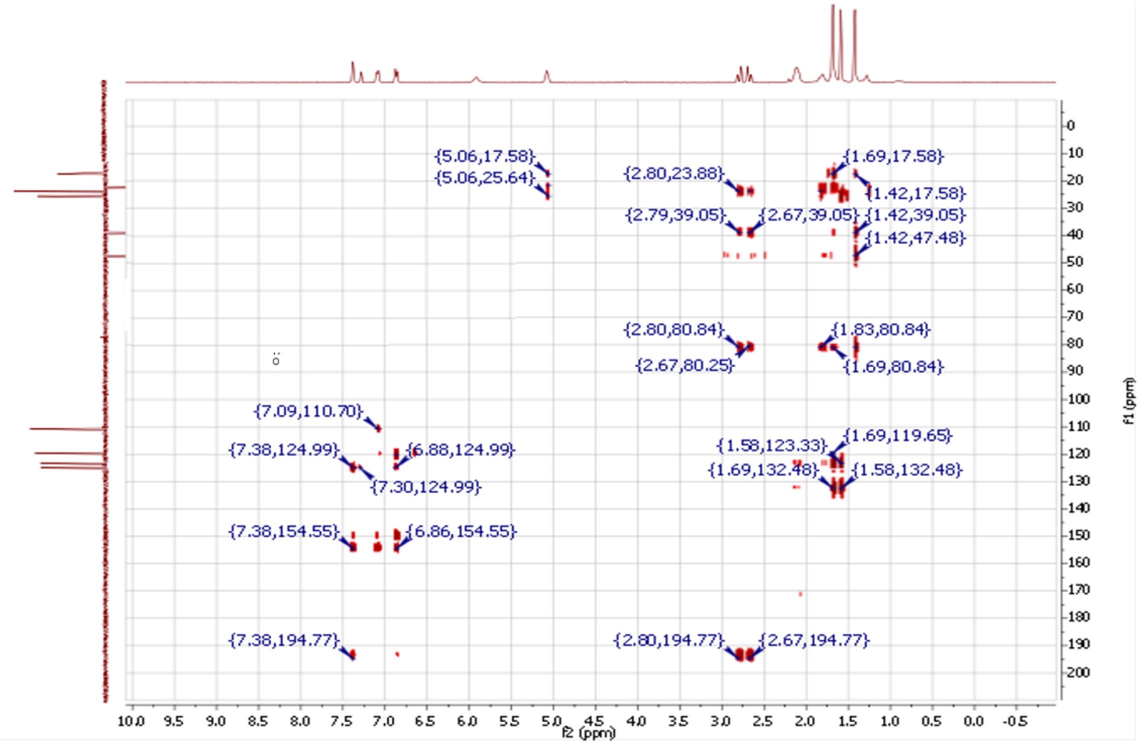

Figure S19. HMBC spectra of compound **6** ($CDCl_3$).

3. Biossays

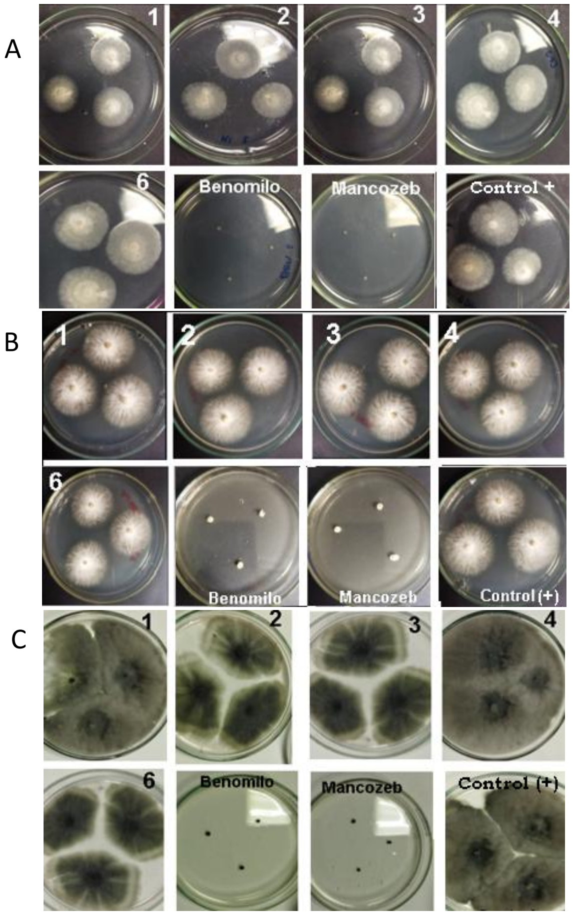

Figure S21. Fungicidal and fungistatic assay of the compounds with the greatest potential to inhibit the mycelial growth of **A**. *F. solani*, **B**. *M. roreri* and **C.** *L. theobromae*. Control (+): Blank of the IMG assay.

plants

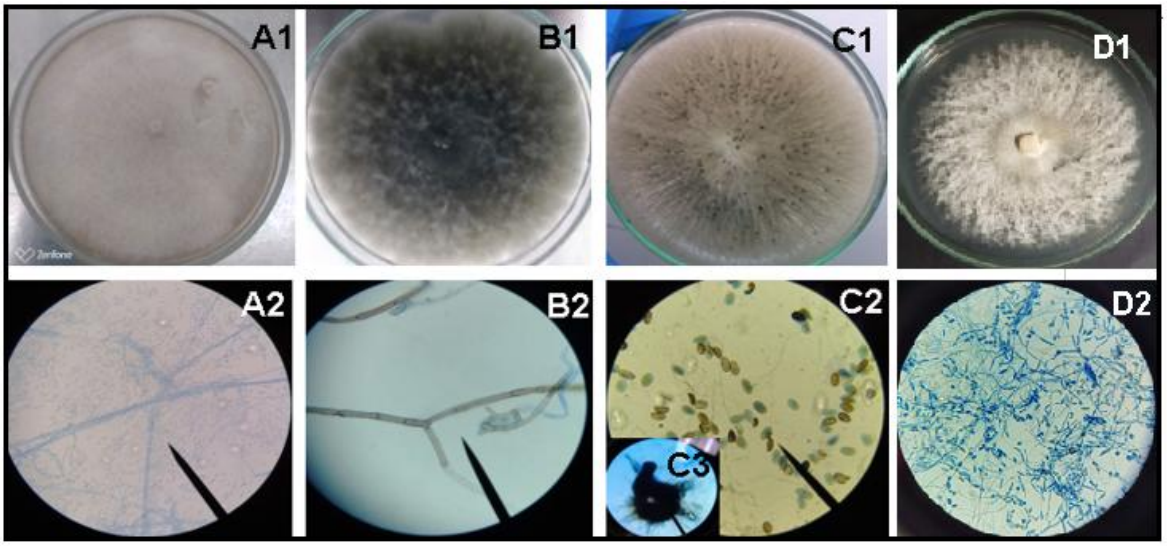

Figure S20. Strains of phytopathogenic fungi used in the bioassays: **A1)** *F. solani* strain in PDA; **A2)** Microscopy of *F. solani* on PDA**; B1)** *L. theobromae* strain in PDA; **B2)** Microscopy of *L. theobromae* on PDA; **C1)** *L. theobromae* strain on maltose-casamino acid agar medium, **C2)** *L. theobromae* strain on maltose-casamino acid agar medium; **C3)** Pycnidium of *L. theobromae*; **D1)** *M. roreri* strain on PDA and **D2)** Microscopy of *M. roreri* on PDA. (Author's own).
